# Decoupling Mechanical Confinement and Fibrotic Extracellular Matrix Signaling in Vestibular Schwannoma Using Tunable 3D Hydrogels

**DOI:** 10.1007/s12195-026-00911-3

**Published:** 2026-05-15

**Authors:** Melanie Fisher, Han TN. Nguyen, Rinky Ghosh, Yael Vodovotz, Yin Ren

**Affiliations:** 1 Division of Otology, Neurotology, and Cranial Base Surgery, Department of Otolaryngology - Head and Neck Surgery, The Ohio State University Wexner Medical Center, Columbus, OH USA; 2 Department of Food Science and Technology, The Ohio State University College of Food, Agricultural and Environmental Sciences, Columbus, OH USA

**Keywords:** Acoustic neuroma, Vestibular schwannoma, YAP, ECM remodeling, 3D hydrogel, Stiffness

## Abstract

**Purpose:**

Vestibular schwannoma (VS) progressively stiffens and remodels its extracellular matrix (ECM) during growth. However, how mechanical confinement and adhesive ECM signaling regulate schwannoma behavior *in vitro* remain incompletely defined.

**Methods:**

Human Nf2^-/-^ schwannoma and primary VS cells established from fresh surgical specimens were cultured in complementary 3D hydrogel platforms. Biochemically inert agarose hydrogels spanning physiologic to pathologic stiffnesses created a non-adhesive mechanical confinement environment, while type I collagen hydrogels modeled an adhesive environment with a dense, fibrillar matrix characteristic of fibrotic tumor ECM. Hydrogel stiffness was quantified by rheology. Cell viability, proliferation, morphology, mechanosensitive signaling, and ECM remodeling were quantified. Mechanical stress was relieved by enzymatic degradation. YAP activity was pharmacologically inhibited, and transforming growth factor-β (TGF-β) was used to induce collagen remodeling.

**Results:**

Under non-adhesive confinement, increasing stiffness suppressed schwannoma reduced cell spreading, decreased N-cadherin expression, and increased nuclear YAP localization. Stress relief reversed YAP activation while enabling enhanced proliferative recovery and increased N-cadherin–associated adhesion. Cells under increased confinement exhibited increased sensitivity to YAP inhibition, indicating confinement-dependent reliance on mechanotransduction. In contrast, adhesive ECM conditions supported active matrix remodeling with increasing stiffness, including elevated activities of MMP9 and phosphorylated focal adhesion kinase (pFAK). TGF-β induced both collagen disorganization and SMAD3 nuclear localization, which was attenuated by YAP inhibition.

**Conclusions:**

Mechanical confinement and ECM composition drive distinct, context-dependent adaptation programs in VS. As stiffness increases, cells in non-adhesive environments adopt a reversible, YAP-associated stress response, while an adhesive ECM shifts behavior toward matrix remodeling and cell adhesion-driven signaling.

**Supplementary Information:**

The online version contains supplementary material available at 10.1007/s12195-026-00911-3.

## Introduction

Vestibular schwannoma (VS) is one of the most common brain tumors arising from Schwann cells of the eighth (cochleovestibular) cranial nerve [1, 2], leading to progressive hearing loss, tinnitus, and vertigo. Large VS causes brainstem compression and hydrocephalus, resulting in substantial neurological morbidity and even death [1]. A clinically important subset of VS exhibits aggressive behavior characterized by rapid tumor growth, adherence to surrounding cranial nerves and brainstem, and increased tumor stiffness. These features complicate surgical resection and are associated with worse clinical outcomes [3–5]. Understanding the biological factors that underlie these aggressive phenotypes remains critical for VS management.

Tumor progression is accompanied by remodeling of the tumor microenvironment, including increased deposition and remodeling of extracellular matrix (ECM), altered matrix organization, and development of fibrosis [6, 7]. Collagen matrices increase tissue stiffness and regulate tumor cell behavior through integrin-dependent adhesion signaling and mechanotransduction pathways, including focal adhesion kinase (FAK) activation and YAP/TAZ-mediated transcriptional responses [8–10]. Across multiple cancer types, fibrotic ECM is characterized by elevated collagen content and disorganized collagen fiber architecture and is associated with increased firmness, invasive behavior, immune modulation, and resistance to chemotherapy [11–14]. VS exhibits increased collagen deposition and stromal remodeling, suggesting that biomechanical changes in ECM are associated with disease progression [4, 15]. However, whether stiffness-associated schwannoma behavior reflects physical confinement, adhesive ECM signaling, or their combined effects remain unclear.

Importantly, VS demonstrates a unique mechanical trajectory during growth. Tumors originate within bony confines of the internal auditory canal (IAC) and subsequently extend into the more compliant cerebellopontine angle (CPA), exposing tumor cells to dynamic changes in physical confinement and ECM context [2]. This progression implies that schwannoma cells must adapt not only to increasing matrix stiffness but also to evolving degrees of confinement and adhesive matrix engagement. In vivo, however, tissue stiffness is inseparable from changes in ECM composition, ligand density, degradability, and dimensionality, making it difficult to isolate the independent contributions of mechanical confinement versus adhesive remodeling to schwannoma behavior.

Conventional two-dimensional (2D) cell cultures fail to recapitulate the three-dimensional (3D) confinement and compressive stress states experienced by tumor cells *in vivo*, while many 3D culture systems confound mechanical stiffness with biochemical cues [16]. Engineered 3D hydrogel systems provide a powerful strategy to decouple these variables. Inert hydrogels such as agarose permit precise tuning of stiffness while minimizing adhesive interactions. In contrast, collagen-based hydrogels introduce adhesive, fibrillar ECM components that support cell–matrix interactions, adhesion signaling, and matrix remodeling. Thus, comparing these complementary platforms allows us to isolate the effects of matrix stiffness from ECM composition and assess distinct cellular responses.

In this study, we developed tunable 3D agarose and collagen hydrogels to examine how matrix stiffness and ECM composition independently regulate schwannoma behavior. Using agarose gels spanning physiologic to pathologic stiffnesses, we isolated the effects of non-adhesive mechanical confinement on cell viability, proliferation, and mechanotransductive signaling. We then assessed whether stiff confinement induced a YAP-dependent stress-adaptation state that increased sensitivity to YAP inhibition. In parallel, we developed collagen hydrogels to investigate adhesion-dependent signaling, ECM remodeling, and fibrosis. Finally, we examined how TGF-β induced YAP-dependent collagen reorganization under mechanical confinement. Together, these hydrogel systems provide a mechanically defined framework to study how stiffness and ECM context shape tumor adaptation, ECM remodeling, and therapeutic vulnerability in VS.

## Methods

### Cell Culture

Primary VS culture was established from surgically resected tumor specimens obtained under institutional review board approval from the Ohio State University (IRB#1994H0241) using published protocols [17–19]. VS specimens were rinsed in PBS, dissected into < 2mm^3^ chunks and treated with collagenase in Dulbecco’s Modified Eagle Medium (DMEM) with Hams’ F12 mixture, 10% fetal bovine serum (FBS), 1% Penicillin/Streptomycin and 1% Glutamax (Life Technologies). Cells were passaged when confluency reached over 80%. Human Nf2^-/-^ VS cells (HEI193), originally derived from a patient with neurofibromatosis type 2 [20], were maintained in DMEM supplemented with 10% FBS and 1% penicillin–streptomycin. Cells were cultured at 37 °C with 5% CO₂.

## Hydrogel Fabrication

### 3D Agarose Hydrogels

Low gelling temperature agarose (Sigma-Aldrich, A9414) was dissolved in PBS by heating in short intervals until fully solubilized. Agarose solutions were prepared at final concentrations of 0.35%, 0.5%, 1%, and 4% (w/v). Following dissolution, agarose was cooled to 37 °C prior to cell encapsulation. Cell suspensions were gently mixed into the agarose solution to ensure uniform distribution. Gels were prepared and allowed to solidify at room temperature for 10–30 minutes before the addition of DMEM.

### 2D and 3D Collagen Hydrogels

Collagen matrices were prepared using FibriCol, type I bovine collagen (Advanced BioMatrix, 5133) per manufacturer’s protocol. Chilled collagen was neutralized by combining eight parts collagen with one part 10X PBS, followed by pH adjustment to 7.2–7.6 using sterile 0.1 M NaOH, and maintained at 2–10 °C. For 3D collagen cultures, cells were suspended in the neutralized collagen solution prior to solidification and polymerized at 37 °C for 90 minutes before being overlaid with complete culture medium. For 2D collagen cultures, neutralized collagen solutions were polymerized on tissue culture plastic under identical conditions, after which cells were seeded directly onto the collagen-coated substrates.

## Rheology Storage Modulus

Rheological measurements were performed to determine the storage modulus (G’) of agarose and collagen hydrogels. Cell-laden hydrogels (1 mL per well) were prepared in 24-well plates at a seeding density of 50,000 cells/mL. Baseline agarose and collagen hydrogels were measured at Day 1 following gel formation, whereas TGF-β–treated collagen gels were measured at day 7. Measurements were conducted using a Discovery HR-2 rheometer (TA Instruments) equipped with a 20 mm parallel plate geometry. All tests were performed at 37 °C. Oscillatory frequency sweep tests were performed at a constant strain of 1.0%, selected from the linear viscoelastic regime based on preliminary amplitude sweep tests, over a frequency range of 0.1–2.0 Hz. The storage modulus (G’) was recorded across the frequency range, and stiffness values were defined as G’ measured at 1 Hz. The same measurement protocol was applied to agarose, collagen, enzymatically degraded agarose gels, and TGF-β–treated collagen gels.

## Cell Viability and Proliferation

Cell viability in 3D agarose hydrogels was assessed using a ViaStain AOPI (Revvity Health Sciences, CS2-0106-5ML). Agarose gels were prepared in 24-well plates using 500 µL of cell-laden hydrogel per well at a 200,000 cells/mL and cultured for either 1 or 7 days. At each point, culture medium was removed and gels were incubated with AOPI in 1 × PBS (15 µL per well) at 37 °C for 30 minutes. Gels were fixed with 4% PFA, removed from wells, mounted onto glass slides, and cover-slipped using fluorescence mounting medium. Imaging was performed using an Olympus FV3000 laser scanning confocal microscope with a 40x objective. Z-stack images were acquired and converted to maximum intensity projections. Live and dead cells were identified based on green (488 nm) and red (594 nm) fluorescence, respectively, and viability was quantified using Fiji (ImageJ) by thresholding and particle analysis (>60 µm^2^). Percent viability was calculated as the number of live cells divided by the total number of cells [21]. Cell proliferation was quantified using CellTiter-Glo 3D Cell Viability Assay (Promega, G9683) per manufacturer’s instructions. Cells were embedded in hydrogels at an initial density of 10,000 cells/mL in 50 µL gel per well of a 96-well plate with 50 µL of culture medium. Assay reagent was added at designated time points and luminescence was measured and normalized to cell-free gel background (BioTek Cytation 1, Agilent Technologies). Signal was normalized to Day 1 measurements.

## Immunofluorescence Staining and Analysis

Immunofluorescence staining was performed on hydrogels in 24-well plates at 250 µL per well with a seeding density of 50,000 cells per well. Gels were fixed with 4% paraformaldehyde, permeabilized with 0.05% Triton X-100, and blocked with donkey serum. Primary antibodies were incubated overnight at 4 °C in the dark. Antibodies used included N-cadherin (Proteintech, 22018-1-AP, 1:200), YAP (Abcam, ab56701, 1:200), pFAK (Cell Signaling Technology, Y397, 1:100), MMP9 (R&D Systems, AF911, 1:100), SMAD3 (Cell Signaling Technology, 9523, 1:200), OCT6 (Abcam, ab27925, 1:200), SOX10 (Novus, NBP2-44475, 1:200). Appropriate fluorescent secondary antibodies were used for 1 hour at room temperature (1:500) and gels were counterstained with DAPI. Cell cluster morphology was quantified by identifying DAPI-stained nuclei and defining multicellular clusters, followed by particle-based area measurements of cluster area. Mean fluorescence intensities were thresholded across groups to remove background. N-cadherin intensity was normalized to number of nuclei per region of interest. Nuclear-to-cytoplasmic (N/C) ratios of YAP and SMAD3 were calculated by defining nuclear ROIs. Cytoplasmic regions were generated by subtracting nuclear from the total cellular region. All analyses included at least 5 randomly selected ROIs.

## RNA Extraction and Gene Expression via RT-qPCR

Cells were embedded in agarose or collagen hydrogels at 2 × 10⁶ cells/mL and cultured for 3 days. Gels were lysed in TRIzol (Invitrogen 15596026), and RNA was purified using Qiagen silica column–based kit (74104) according to Manufacturer’s protocol. cDNA was synthesized using iScript cDNA Synthesis Kit (Bio-Rad 1708890) and RT-qPCR was performed using iTaq Universal SYBR Green Supermix (Bio-Rad 1725121) on a real-time PCR system. Relative gene expression was calculated using the ΔΔCt method and normalized to GAPDH. Gene expressions in 3D collagen gels were normalized to cells cultured on 2D collagen substrates using the same concentration. For agarose experiments, gene expression was normalized to cells cultured in 0.35% agarose gels. Primer sequences used for RT-qPCR are listed in Table S1.

## Enzymatic Stress Relief Using Agarase

Agarose hydrogels were enzymatically degraded using agarase (Thermo Fisher Scientific, FEREO0461) to remove mechanical confinement. Cells were cultured in agarose gels for 72 hours, removed from the wells, and incubated in agarase diluted in TAE buffer (5 U/mL) at 37 °C for 30 minutes with gentle inversion every 10 minutes and periodic pipetting. Cells were pelleted by centrifugation, washed with PBS, and re-embedded in agarose gels of the indicated stiffness and cell density. Time-matched cultures in their original stiffness hydrogels were included as controls.

## Verteporfin Treatment

Cells embedded in hydrogels were treated with verteporfin (MedChemExpress, CL-318952) diluted in DMEM at specific concentrations for 24 hours. Downstream effects were assessed by cell viability and immunofluorescence analyses as described above.

## TGF-β Treatment and PSR/CT-FIRE Analysis

To induce fibrotic extracellular matrix remodeling, cells are embedded in 3D collagen hydrogels and treated with recombinant human TGF-β in DMEM (PeproTech, 10021C10UG) at 10 ng/mL for the indicated durations. Changes in collagen organization, gene expression, and SMAD3 nuclear localization were quantified as described previously. Where indicated, cultures were co-treated with verteporfin. Collagen organization was assessed by picrosirius red (PSR) staining (Abcam, ab150681) followed by polarized light microscopy as described previously [15]. Gels were fixed in 4% formaldehyde, cryoprotected in 30% sucrose, embedded in OCT compound, and sectioned at 10 µm for staining. PSR-stained sections were imaged under brightfield and polarized light using identical acquisition settings. Polarized images were analyzed using CT-FIRE (Laboratory for Optical and Computational Instrumentation, University of Wisconsin) to quantify collagen fiber orientation as described [15], defined as the percentage of fibers oriented outside ± 20° of the median fiber angle. Quantification was performed across at least 5 independently selected ROIs per condition.

## Statistical Analysis

Statistical analyses were performed using GraphPad Prism (San Diego, CA). Two-group comparisons were assessed using Mann-Whitney U tests. For comparisons involving more than two groups, one-way or two-way ANOVA were used where appropriate, followed by Tukey’s or Sidak’s multiple comparisons tests. Non-parametric Kruskal–Wallis tests with Dunn’s post hoc comparisons were used for selected image-based analyses. For gene expression panels involving multiple comparisons, multiple unpaired t tests with false discovery rate (FDR) correction were applied. Statistical significance was defined as *p* < 0.05 unless otherwise indicated.

## Results

**Fig. 1:**
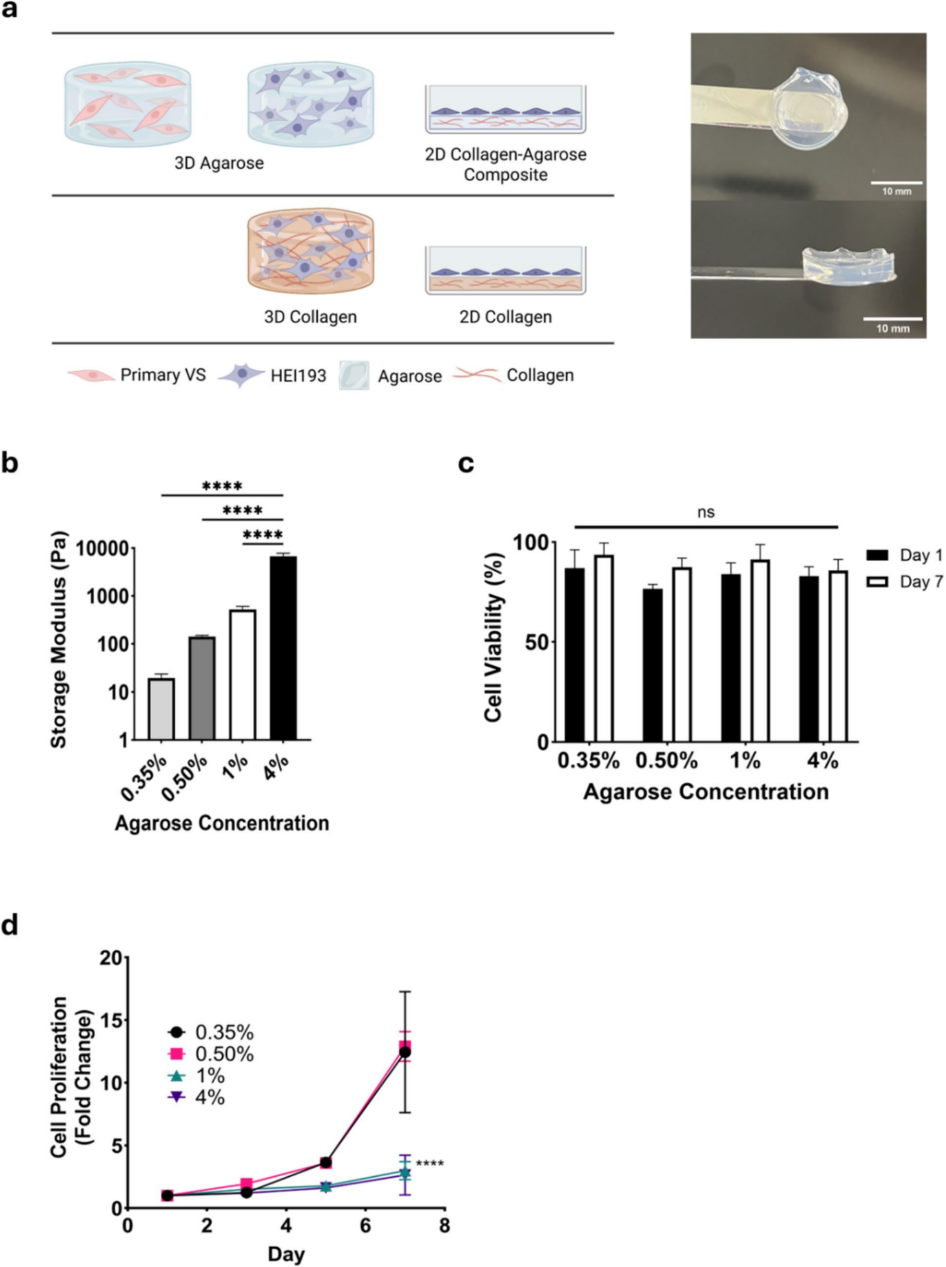
Tunable 3D agarose hydrogels support schwannoma proliferation **a** Schematic overview of 3D and 2D cell schwannoma culture platforms using agarose, collagen, and collagen-agarose composite hydrogel systems. Right, representative photographs of a 3D agarose hydrogel (1%). Scale bars are 10 mm. **b** Storage modulus (Pa) of agarose hydrogels increases with agarose concentration (0.35–4%). N = 3 independent replicates, ****p < 0.0001 by one-way ANOVA with Tukey’s post hoc analysis. **c** Viability of HEI193 cells in agarose hydrogels on Day 1 and 7. ns, not significant, by two-way ANOVA with Sidak’s post hoc. d Proliferation of HEI193 cells in agarose hydrogels over 7 days, ****p < 0.0001 by two-way ANOVA

To investigate how mechanical confinement regulates VS biology, we embedded human Nf2^-/-^ schwannoma cells (HEI193) within biochemically inert 3D agarose hydrogels of tunable stiffnesses (Fig. 1a). We chose agarose to isolate biophysical effects on cellular behavior independent of adhesive or biochemical cues. Agarose concentrations from 0.35 to 4% yielded storage moduli from 20 Pa to 6.7 kPa (Fig. 1b), spanning the physiological stiffness of soft neural tissue such as brain (100–1,000 Pa), and extending into pathological range characteristic of fibrotic tumors (>1 kPa) [6, 7, 22]. The agarose hydrogels remained stable for > 7 days and were fully compatible with standard culture media and workflows.

We next assessed the effect of hydrogel stiffness on schwannoma survival and proliferation. Cells remained > 80% viable across all stiffnesses on day 7, with no significant differences between groups (Fig. 1c; Fig. S1a), indicating that increasing confinement did not compromise cell survival. In contrast, cell proliferation demonstrated stiffness-dependent suppression: by Day 7, cells in stiff matrices (1% and 4% hydrogels) exhibited reduced proliferation compared to those in soft 3D matrices (0.35% and 0.5%, *p* < 0.0001) (Fig. 1d). Additionally, given the preserved viability across conditions, this reduction in proliferation is unlikely to be driven by limitations in nutrient or oxygen availability, consistent with prior studies using agarose hydrogels with similar concentrations [21, 23]. Therefore, although schwannoma cells tolerated a broad range of matrix stiffness, elevated confinement diminished their proliferative capacity, consistent with a shift towards mechanical stress-adaptation and pro-survival signaling [21, 24, 25]. Together, these results establish a reproducible and physiologically relevant 3D model where matrix stiffness regulates schwannoma cell behavior.

**Fig. 2:**
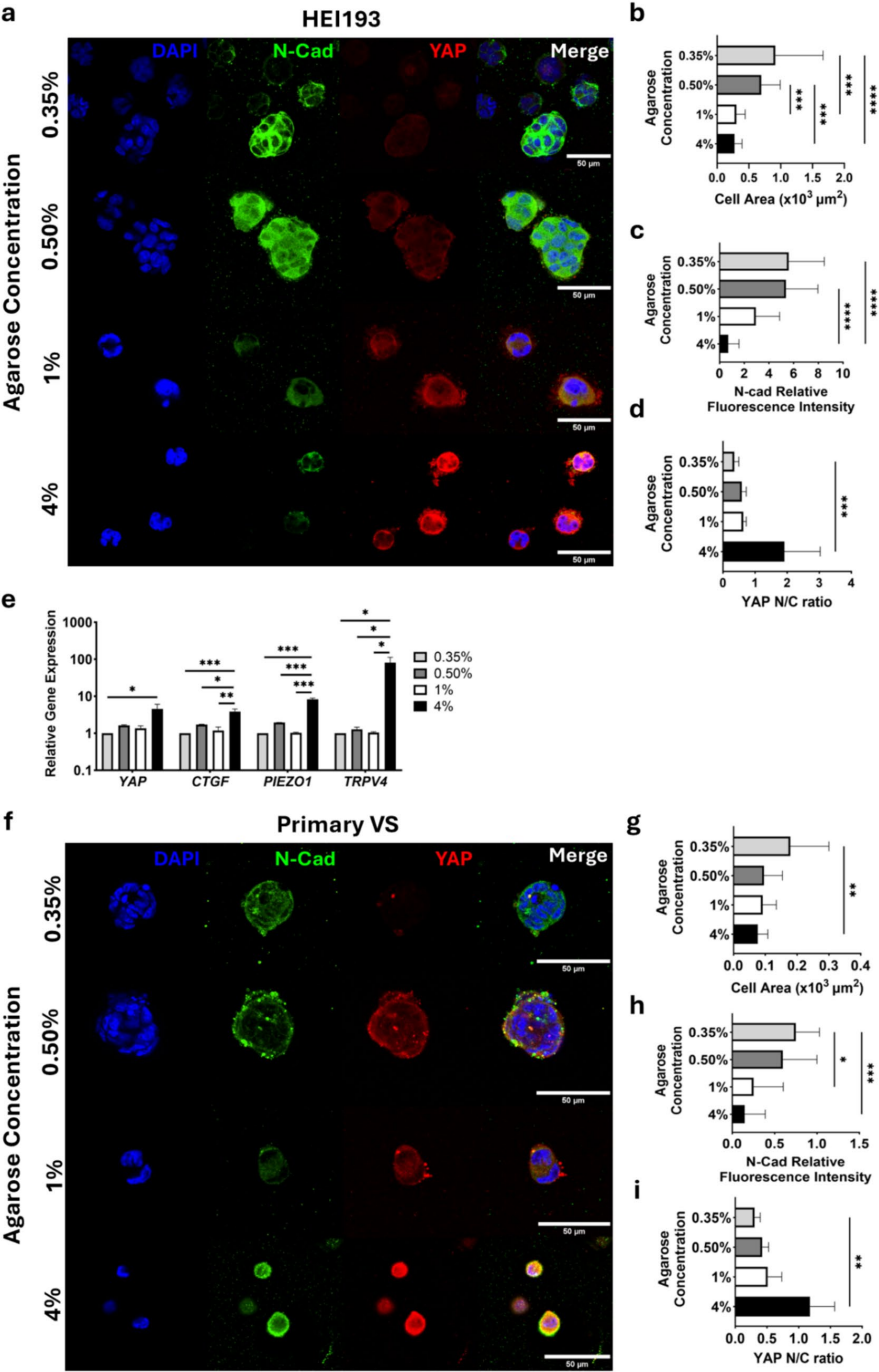
Matrix stiffness regulates morphology and mechanosensitive signaling in 3D agarose schwannoma cultures **a** Representative immunofluorescence of HEI193 cells embedded in 3D agarose hydrogels of increasing stiffness (0.35–4%) stained for nuclei (DAPI, blue), N-cadherin (green), and YAP (red). Scale bars are 50 µm. **b–d** Quantification of HEI193 cell cluster area **(b)**, N-cadherin mean fluorescence intensity **(c)** and YAP N/C ratio **(d)** across agarose concentrations,***p < 0.001, ****p < 0.0001 by Kruskal-Wallis test with Dunn’s post hoc. **e** qRT-PCR quantification of normalized mRNA expression of *YAP*, *CTGF*, and *PIEZO1* and *TRPV4* in HEI193 cells across agarose concentrations, *p < 0.05, **p < 0.01, ***p < 0.001 by one-way ANOVA with Tukey’s post hoc. **f** Representative immunofluorescence images of primary VS cells embedded in 3D agarose hydrogels stained for nuclei (DAPI), N-cadherin, and YAP. Scale bars are 50 µm. **g–i** Quantification of cell cluster area (**g**), N-cadherin relative mean fluorescence intensity **(h)**, and YAP N/C ratio **(i)** in primary VS cultures. N = 3 hydrogels, *p < 0.05, **p < 0.01, ***p < 0.001 by Kruskal-Wallis test with Dunn’s post hoc test

Having established a 3D system to study mechanical regulation of schwannoma cell behavior, we next investigated how extrinsic mechanical cues influence cell morphology, adhesion, and mechanosensitive signaling (Fig. 2a). Cells encapsulated in soft agarose gels formed large, spread-out clusters, whereas increasing stiffness resulted in smaller and compact cell aggregates. Quantitative analysis confirmed a marked reduction in cluster area with increased stiffness (915.5 μm^2^ in 0.35% vs. 273.6 μm^2^ in 4% agarose; Fig. 2b). Since adherens junction proteins (N- and E-cadherin) and integrins regulate cell–cell and cell–matrix adhesion [26], we examined whether N-cadherin represented a mechanosensitive adhesion marker linked to increased invasiveness. N-cadherin fluorescence intensity was significantly reduced with increased stiffness, exhibiting an 8-fold reduction in 4% compared to 0.35% agarose (*p* < 0.0001) (Fig. 2c). Although a stiffer microenvironment is often associated with N-cadherin upregulation in other tumor types [27–29], its reduction here suggests a schwannoma-specific adaptation to mechanical confinement. In the high-stiffness, non-adhesive environment, reduced N-cadherin mediated adhesion reflects decreased cell–cell adhesion under mechanical confinement, favoring adhesion-independent mechanotransduction pathways such as YAP to tolerate restrictive mechanical stress. Notably, although proliferation was similarly suppressed in both 1% and 4% agarose conditions (Fig. 1d), mechanosensitive outputs including YAP activation and gene expression continued to increase with stiffness. This divergence suggests that proliferation is limited by loss of adhesion, as indicated by decreased N-cadherin expression, and confinement at intermediate stiffness. Further increases in stiffness continue to enhance mechanosensitive signaling without producing additional effects on cell growth. These findings support a confinement-adapted phenotype in which reduced adhesion coincides with decreased proliferation and increased mechanosensitive signaling, which has been associated with enhanced tumorigenic potential in other cancer models [24, 25, 30].

YAP, a key mechanosensitive transcriptional co-activator, integrates matrix stiffness and cytoskeletal tension to drive pro-survival and invasive transcriptional programs [31, 32]. Accordingly, we quantified the YAP nuclear-to-cytoplasmic (N/C) ratio as a mechanotransduction readout [8]. YAP nuclear localization was significantly elevated in 4% compared to 0.35% gels (N/C ratio 1.9 vs 0.35, *p* = 0.0004) (Fig. 2d). Primary VS cultures exhibited similar stiffness-dependent trends in cell morphology and expression of N-cadherin and YAP (Fig. 2f–i), validating the translational relevance of the 3D model. Consistent with stiffness-enhanced YAP signaling, expression of *YAP* and its downstream effector *CTGF* increased 4.5-fold (*p* = 0.035) and 3.8-fold (*p* = 0.0069), respectively, in 4% compared to 0.35% hydrogels. Upstream mechanosensitive ion channels were also upregulated, with *PIEZO1* increased by 8.2-fold (*p* < 0.0001) and *TRPV4* by 81-fold (*p* = 0.027) (Fig. 2e). Together, these data show that matrix stiffness enhances Schwannoma cell mechanotransduction via coordinated activities of mechanosensitive ion channels and YAP.

**Fig. 3:**
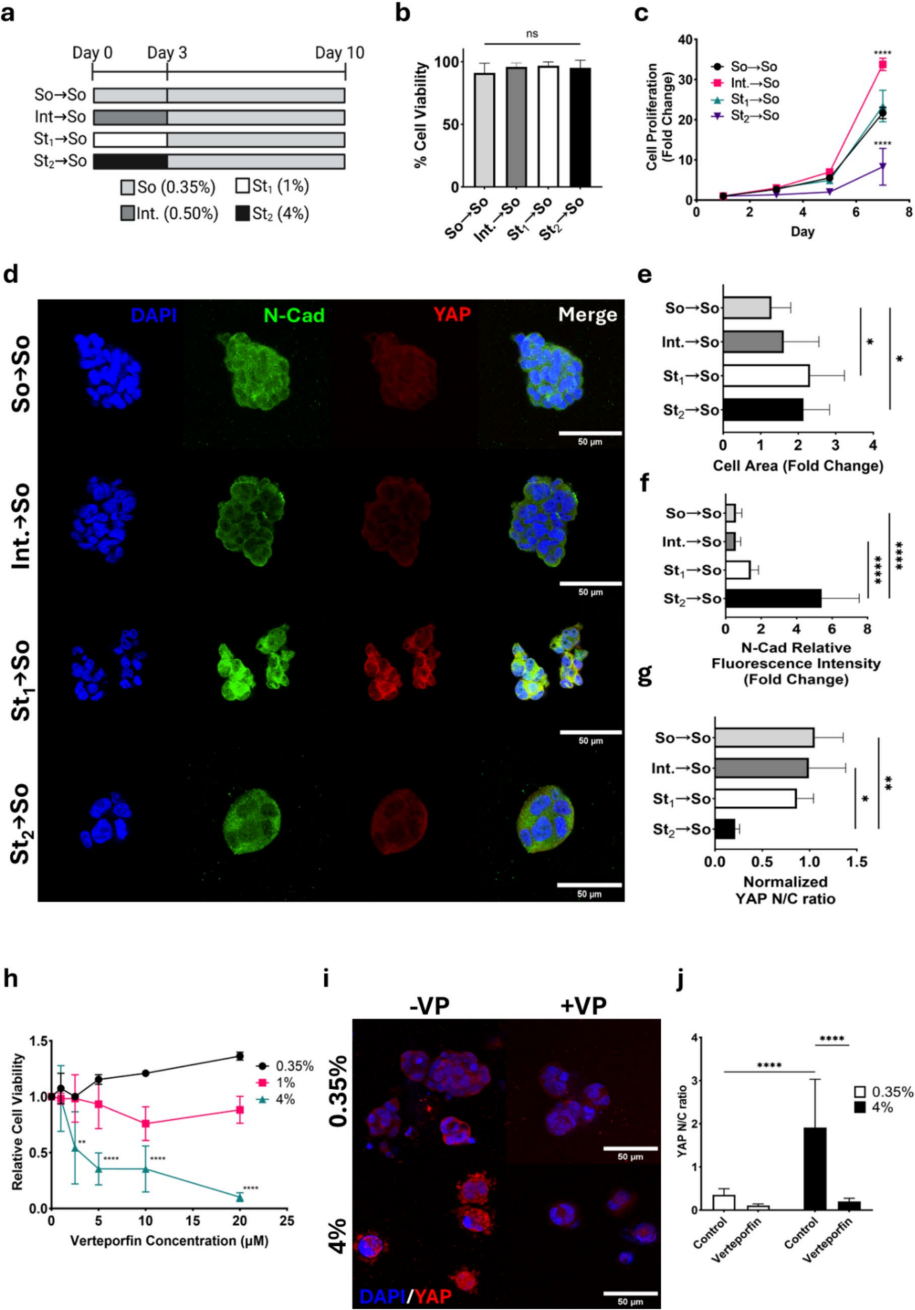
Stress relief reverses stiffness-induced phenotypes, while YAP inhibition targets stiffness-primed schwannoma cells **a** Schematic showing 3D culture in agarose hydrogels of defined stiffness, followed by agarase–mediated stress relief and re-embedding in soft hydrogels. (0.35% agarose, Soft [So]; 0.50%, Intermediate [Int]; 1%, Stiff 1 [St₁]; 4%, Stiff 2 [St₂]). **b** Cell viability following stress relief. ns, not significant, by one-way ANOVA with Tukey’s post hoc. **c** Cell proliferation following stress relief, plotted as fold change relative to Day 1 and normalized to stiffness-matched controls. ****p < 0.0001 by two-way ANOVA with Tukey’s post hoc. **d** Representative immunofluorescence of HEI193 cells following stress relief. Nuclei (DAPI, blue), N-cadherin (green), and YAP (red). Scale bars are 50 µm. **e–g** Quantification of post-stress relief changes in normalized cell cluster area **(e)**, N-cadherin fluorescence **(f)**, and YAP N/C ratio **(g)**, normalized to time- and stiffness-matched controls. N = 3 hydrogels, *p < 0.05, **p < 0.01, ****p < 0.0001 by Kruskal-Wallis test with Dunn’s post hoc. **h** Relative cell viability of HEI193 cells embedded in agarose hydrogels of varying stiffness (0.35%, 1%, and 4%) treated with verteporfin (VP, 0-20 µM). N = 3 hydrogels, **p < 0.01, ****p < 0.0001 by two-way ANOVA with Tukey’s post hoc. **i** Representative immunofluorescence of HEI193 cells embedded in soft (0.35%) or stiff (4%) agarose hydrogels with or without VP treatment, stained for nuclei (DAPI, blue) and YAP (red). Scale bars are 50 µm. **j** Quantification of YAP N/C ratio following VP treatment. N = 3 hydrogels, ****p < 0.0001 by two-way ANOVA with Sidak’s post hoc test

Given that stiff non-adhesive confinement induced mechanotransduction programs in VS, we next asked whether these responses are reversible upon stress relief. Because cancer cells may exhibit either rapid reversibility or persistent “mechanical memory” [24, 33–38], we tested whether Schwannoma cells retain stiffness-induced changes in proliferation and morphology. HEI193 cells cultured in agarose gels of various stiffnesses (0.35% agarose, Soft [So]; 0.50%, Intermediate [Int]; 1%, Stiff 1 [St₁]; 4%, Stiff 2 [St₂]) were enzymatically digested with agarase and re-embedded in soft agarose hydrogel (0.35% So) for 7 more days (Fig. 3a schematic). Rheometry confirmed agarase-mediated stiffness reduction (Fig. S1b) and cells remained viable (Fig. 3b). Cell proliferation revealed stiffness-dependent recovery following stress relief. Relative to Day 1 post-digestion and stiffness-matched controls, St₁ → So cells fully recovered to So → So levels. Interestingly, Int → So cells exhibited a 33-fold increase in proliferation (*p* < 0.0001), consistent with a potential mechanical priming effect [33, 39]. In contrast, St₂ → So showed a modest 8-fold increase (*p* < 0.001) indicative of partial recovery (Fig. 3c). Therefore, our data suggests that schwannoma growth depends on prior stiffness exposure.

We next assessed the reversibility of stiffness-induced morphological, adhesive, and mechanosensitive signaling changes (Fig. 3d). Post-stress relief measurements were normalized to the corresponding time- and stiffness-matched controls. Stress relief increased cluster area of previously confined cells, with St₁ → So and St₂ → So showing 2.3-fold (*p* = 0.02) and 2.1-fold (*p* = 0.02) increases compared to the 1.4-fold increase in the So → So group, indicating a stronger rebound spreading after confinement in stiffer matrices (Fig. 3e). N-cadherin expression also increased, most notably with the greatest post-relief fold change in St₂ → So cells (5.4-fold), exceeding that of So → So (0.55-fold) and Int → So (0.56-fold) conditions (*p* < 0.0001 for both), demonstrating that greater prior confinement enhanced N-cadherin signaling (Fig. 3f). In contrast, YAP nuclear localization decreased following stress relief. Cells in St₂ → So showed a YAP N/C ratio reduced to 20% and lower YAP activity compared to So → So and Int → So groups (*p* = 0.006 and *p* = 0.025, respectively), consistent with reversibility of YAP activation (Fig. 3g).

We next tested whether mechanically confined schwannoma cells are more sensitive to YAP inhibition by Verteporfin (VP), an inhibitor of YAP–TEAD transcriptional activity by degrading YAP and preventing its nuclear localization [40]. Cell viability in 0.35% gels was preserved at high VP concentrations of 20 µM, whereas cells in 4% agarose gels exhibited dose-dependent reduction in viability, with a 50% reduction at 2.5 µM of VP (*p* = 0.008 and *p* = 0.004 vs. 0.35% and 1%) and a 90% reduction at 20 µM of VP (*p* < 0.0001) (Fig. 3h). To confirm VP effectively inhibited YAP activation, we quantified YAP nuclear localization by immunofluorescence in cells embedded in 0.35% and 4% agarose with or without VP. VP led to a > 9-fold decrease in the YAP N/C ratio compared to untreated controls (*p* < 0.0001, Fig. 3i–j). These results indicate that matrix stiffness increases Schwannoma cell reliance on YAP signaling for survival, thereby rendering them more vulnerable to disruption of YAP-TEAD signaling, highlighting the potential of YAP-targeted therapies in mechanically stiff VS.

**Fig. 4:**
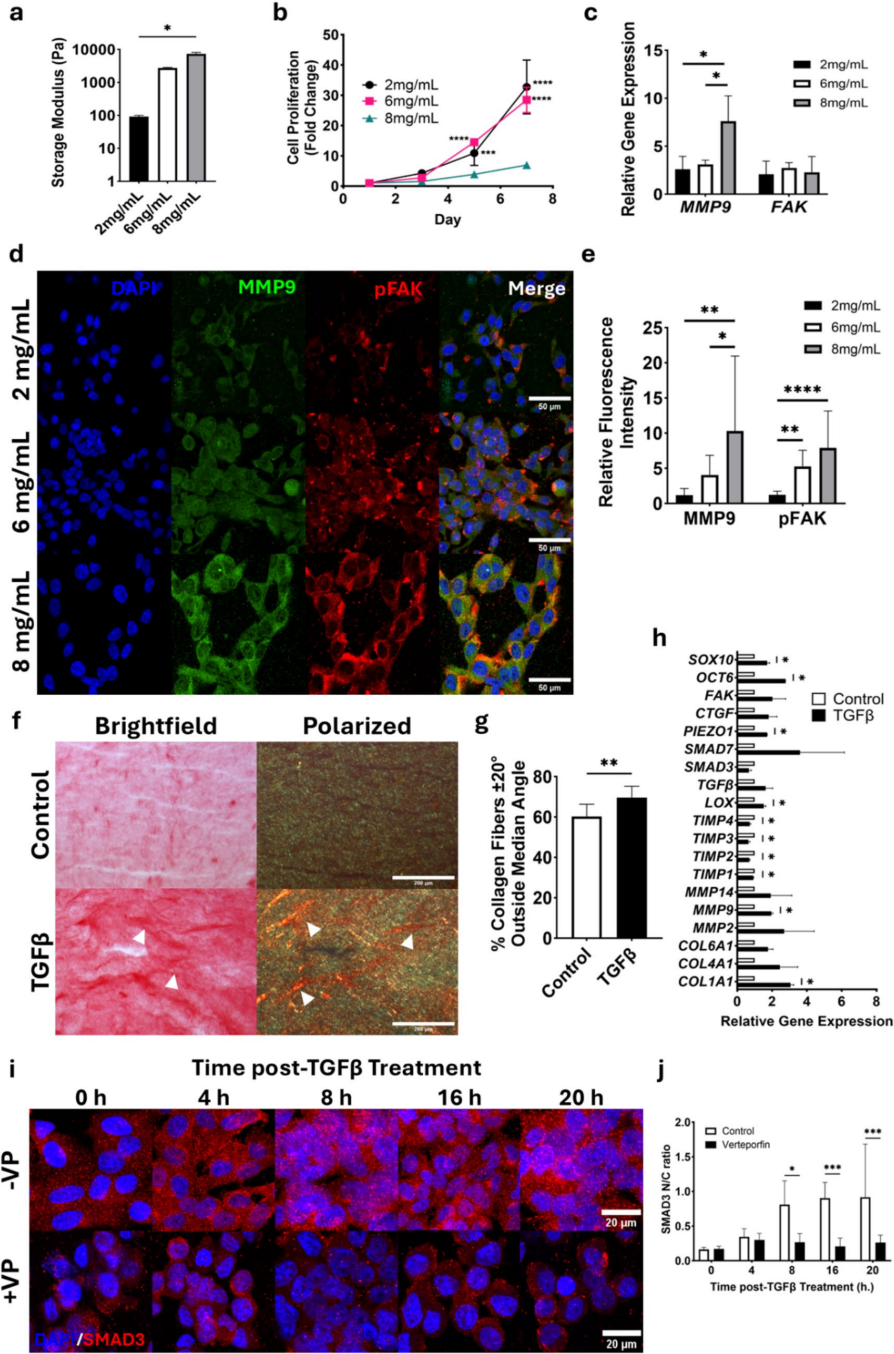
Collagen stiffness and TGF-β promote fibrotic signaling and matrix remodeling in schwannoma cells embedded in 3D collagen hydrogels **a** Storage modulus (Pa) of collagen hydrogels (2–8 mg/mL), *p < 0.05 by Kruskal-Wallis test with Dunn’s post hoc. **b** Proliferation of HEI193 cells embedded in 3D collagen hydrogels over 7 days, showing stiffness-dependent suppression of proliferation. N = 3 hydrogels, ***p < 0.001, ****p < 0.0001 by two-way ANOVA with Tukey’s post hoc. **c** Relative mRNA expression of *MMP9* and *FAK* in HEI193 cells in 3D collagen hydrogels compared to stiffness-matched 2D collagen gels. *p < 0.05 by one-way ANOVA with Tukey’s post hoc. **d**, **e** Representative immunofluorescence images **(d)** and quantification **(e)** of MMP9 and pFAK in HEI193 cells in 3D collagen hydrogels of increasing stiffness. *p < 0.05, **p < 0.01, ****p < 0.0001 by one-way ANOVA with Tukey’s post hoc. Scale bars are 50 µm. **f** Representative brightfield (left) and polarized light (right) images of picrosirius red-stained (PSR) collagen hydrogels following TGF-β treatment. Arrows indicate regions of altered collagen organization in brightfield images and increased red and yellow birefringence under polarized light. Scale bars are 200 µm. **g** Quantification of collagen fiber disorganization following TGF-β treatment, expressed as the percentage of fibers oriented ± 20° outside the median angle. **p < 0.01 by Mann-Whitney test. **h** Relative expression of genes involved in collagen content (*COL1A1, COL4A1, COL6A1),* ECM remodeling (*MMP2, MMP9, MMP14, TIMP1-4, LOX*), mechanotransduction (*PIEZO1, FAK, CTGF*), Schwann cell markers (*OCT6, SOX10*), and fibrosis signaling (*TGFB, SMAD3, SMAD7, CTGF*) with or without TGF-β treatment. *q < 0.05 by multiple unpaired t tests with FDR correction. **i** Representative immunofluorescence images of HEI193 cells treated with TGF-β in the presence or absence of verteporfin (VP) over time (0-20 h), stained for nuclei (DAPI, blue) and SMAD3 (red). Scale bars are 20 µm. **j** Quantification of SMAD3 N/C ratio following TGF-β treatment with or without VP. *p < 0.05, ***p < 0.001 by two-way ANOVA with Sidak’s post hoc analysis

While the 3D agarose hydrogel isolated the effects of mechanical confinement, this system lacked adhesive and degradable matrix components characteristic of native ECM in the tumor microenvironment. To determine how adhesive matrix stiffness regulates Schwannoma behavior, we generated 3D collagen-I hydrogels, which represents one of the most abundant ECM proteins in the microenvironment and a key regulator of tumor progression, spanning physiologic and pathologically stiff conditions [41]. Rheological analysis confirmed a progressive increase in storage modulus from 90 Pa at 2 mg/mL of Col-I to 7.3 kPa at 8 mg/mL (Fig. 4a). Schwannoma cells in 3D collagen exhibited elongated morphologies and increased expression of maturation markers (OCT6 and SOX10) compared to 2D collagen and tissue culture plastic (Fig. S2a–f). Increased collagen stiffness progressively reduced Schwannoma proliferation, with 8 mg/mL collagen gels showing a 78% reduction compared to 2 mg/mL (*p* < 0.001) and a 75% reduction compared to 6 mg/mL conditions at day 7 (*p* < 0.0001; Fig. 4b). However, compared to non-adhesive confinement, cell proliferation in adhesive ECM conditions remained higher at comparable stiffness ranges (Fig. 1d, 4b), where cells in 1% and 4% agarose exhibited more pronounced suppression, indicating that ECM composition may enhance Schwannoma proliferative capacity under mechanical confinement. In contrast, increased stiffness in 2D collagen-agarose substrates enhanced growth, consistent with prior studies [10, 42, 43] (Fig. S2g). Together, these findings indicate that Schwannoma proliferation is highly dependent on both dimensionality and ECM composition.

We next examined stiffness-dependent regulation of adhesion and matrix remodeling genes. *MMP9*, a matrix metalloproteinase that degrades fibrillar collagen, increased with collagen density and was highest in 8 mg/mL Col-I gels (8.7-fold vs. 2 mg/mL, *p* = 0.03 and 2.5-fold vs. 6 mg/mL *p* = 0.05), consistent with other 3D models [44–46]. Because increased collagen density activates focal adhesion signaling [7, 10], we examined focal adhesion kinase (FAK) expression and found that total FAK showed a 2-fold increase compared to stiffness-matched 2D controls (Fig. 4c). Immunofluorescence studies revealed progressive increases in MMP9 and phosphorylated FAK (pFAK) with increased collagen (Fig 4d, e). Furthermore, we evaluated mechanosensitive pathways including mechanosensitive ion channels (*PIEZO1*, *TRPV4*) which exhibited a distinct, non-linear stiffness-dependent regulation, with maximal expression at intermediate collagen stiffness (6 mg/mL) and reduced expression at 8 mg/mL. YAP expression and total YAP signal intensity both increased with collagen stiffness, while the canonical downstream gene CTGF did not significantly change across conditions. CDH2 expression decreased at higher collagen density, whereas N-cadherin protein levels remained relatively unchanged (Fig. S3a–c). These results demonstrate that increasing collagen promotes matrix remodeling and activation of focal adhesion kinase signaling, with mechanosensitive signaling present but not the primary stiffness-associated response.

Transforming growth factor-β (TGF-β) is another key regulator of ECM remodeling and Schwannoma progression [11, 47]. Because TGF-β integrates biochemical and mechanical cues, we examined whether Schwannoma cells embedded within a mechanically relevant 3D collagen matrix exhibit enhanced TGF-β–induced ECM remodeling. Schwannoma cells were embedded in intermediate-stiffness 3D collagen (6 mg/mL) and treated with TGF-β. TGF-β treatment did not alter matrix stiffness (Fig. S3d) but induced alterations in collagen fibril organization: picrosirius red (PSR) staining and polarized light analysis revealed increased red and yellow birefringence, indicative of collagen fiber thickening and bundling, with more heterogeneous fiber orientation in TGF-β–treated gels compared to the predominantly green, uniformly aligned fibers in control gels (Fig. 4f). Collagen disorganization, quantified as the proportion of fibers oriented outside ± 20° of the median fiber angle, also increased in TGF-β–treated gels (*p* = 0.003; Fig. 4g).

TGF-β significantly upregulated ECM remodeling genes involved in collagen production (*COL1A1)*, matrix remodeling (*MMP9)*, collagen crosslinking (*LOX*), the mechanosensitive ion channel *PIEZO1,* and markers of Schwann cell differentiation (*OCT6*, *SOX10*), while downregulating tissue inhibitors of metalloproteinases (*TIMP1–4*) without affecting *FAK*, *CTGF*, *SMAD3*, or *TGFB* (Fig. 4h). Since canonical TGF-β signaling relies on SMAD phosphorylation and nuclear translocation [48], we assessed SMAD3 nuclear localization as a readout of TGF-β activation. In addition, since mechanosensitive pathways such as YAP can further influence TGF-β responses [48, 49], we also examined whether YAP inhibition modulates TGF-β–induced SMAD3 signaling. Under basal conditions, SMAD3 remained largely cytoplasmic. TGF-β treatment induced nuclear localization of SMAD3 over time indicative of sustained activation of canonical TGF-β. Notably, YAP inhibition by VP significantly reduced SMAD3 nuclear localization at 8, 16, and 20 hours compared to TGF-β treatment without VP (*p* = 0.0175, *p* = 0.0005, *p* = 0.001, respectively) (Fig. 4i–j). Together, these results reveal extensive crosstalk between YAP and TGF-β signaling pathways in schwannoma in 3D culture, linking YAP mechanotransduction to matrix remodeling programs under mechanical confinement.

## Discussion

In this study, we developed complementary 3D hydrogel systems to isolate the effects of mechanical confinement and adhesive extracellular matrix signaling in vestibular schwannoma. We demonstrate that non-adhesive confinement suppresses proliferation while promoting YAP signaling that is reversible upon stress relief. Increased stiffness induces reliance on YAP signaling, revealing a stiffness-dependent vulnerability to YAP inhibition. In contrast, an adhesive matrix supports focal adhesion kinase signaling and matrix remodeling under comparable stiffness conditions. Furthermore, TGF-β signaling intersects with YAP to regulate remodeling responses in a mechanically defined 3D environment. This platform provides a means to study VS behavior in a 3D mechanical context that is not captured and cannot be separated by existing in vitro models.

VS progression is accompanied by changes in the mechanical properties of the tumor microenvironment. Our prior work using preoperative magnetic resonance elastography demonstrated that increased tumor stiffness correlated with worse hearing loss, greater likelihood of subtotal tumor resection, and poorer facial nerve outcomes, establishing mechanical stiffness as a clinically relevant feature and potential biomarker of VS progression [4]. In parallel, histologic analysis of human VS specimens revealed increased collagen and altered organization in larger VS, implicating ECM remodeling in tumor progression [15]. However, how mechanical confinement and adhesive matrix remodeling independently shape schwannoma cell behavior *in vitro* remains unclear , as traditional 2D and many 3D culture models cannot decouple mechanical confinement from adhesive ECM signaling [16]. This gap motivates the use of complementary 3D model systems that decouple mechanical confinement from adhesive matrix engagement while preserving relevance to disease progression.

Under mechanical confinement, reduced tumor proliferation alone may be insufficient to define tumorigenic potential. In physically confining environments, restriction of tumor volumetric expansion can suppress proliferation by limiting spheroid growth and inducing cell-cycle arrest, even when cell viability is preserved, as shown in multiple 3D tumor models [24, 50]. Importantly, these growth-restricted states are accompanied by activation of mechanosensitive and stress-responsive signaling pathways rather than loss of aggressive capacity [9]. For example, using 3D collagen spheroid models of human breast cancer, Jahin *et al.* demonstrated that increasing matrix stiffness induces activation of ERK1/2 and YAP despite concurrent suppression of spheroid proliferation and invasion, highlighting that mechanotransduction activation in 3D confined environments does not necessarily translate into traditional pro-tumorigenic functional outputs [25]. The reduced proliferation and cell expansion observed under high stiffness conditions in our models likely reflects this confinement-adapted phenotype rather than diminished tumorigenic potential, consistent with the slow-growing behavior of VS.

YAP is a well-established mechanosensitive transcriptional co-activator that integrates physical cues such as matrix stiffness, confinement, and cytoskeletal tension into transcriptional responses across multiple solid tumors [8, 31]. In confined 3D settings, YAP nuclear localization is frequently observed in mechanically restrictive environments, where physical confinement favors stress-adaptive signaling programs that support tumor progression and aggressiveness in cancer models [25, 31, 43, 51]. In our system, YAP activation under non-adhesive confinement indicates that mechanical stiffness alone is sufficient to drive YAP-dependent stress signaling, independent of ECM adhesion. The concurrent induction of mechanosensitive ion channels, including PIEZO1 and TRPV4, further supports a confinement-driven signaling response consistent with membrane tension–mediated regulation of YAP [52, 53]. Additionally, proliferative output does not scale with mechanosensitive activation, indicating that these responses reflect adaptation to confinement rather than increased growth. These findings indicate that mechanical confinement can actively condition schwannoma cells into a YAP-dependent, stress-tolerant state.

Mechanical memory has been increasingly recognized as a regulator of tumor cell behavior in solid cancers that experience dynamic changes in stiffness and solid stress [34, 36–38]. Experimental studies have shown that exposure to stiff microenvironments can sustain mechanosensitive signaling through nuclear localization of YAP, even after cells are transferred to softer substrates [39, 54]. In glioblastoma and breast cancer models, this sustained mechanotransduction has been associated with persistent invasive and migratory behavior following stiffness conditioning [38, 55]. However, accumulating evidence also indicates that stiffness-induced mechanotransduction can be dynamically regulated. In 3D gastric cancer models, Jang *et al.* showed that matrix softening reversed YAP nuclear localization and integrin cytoskeletal signaling, while phenotypic recovery occurred over a delayed timescale, indicating that past mechanical exposure influenced post-relief behavior [35]. Consistent with these observations, stress relief in our system rapidly normalized YAP activity, while prior confinement conditioned the post-release phenotype toward increased spreading and adhesion. This priming behavior mirrors the mechanical trajectory experienced by VS as tumors extend from the rigid IAC into the softer CPA region, supporting the translational relevance of this model.

Current treatments for VS remain severely limited, with microsurgery and stereotactic radiation representing primary clinical interventions, albeit with significant associated morbidities [1]. Bevacizumab, a monoclonal antibody targeting VEGF, has demonstrated clinical benefit in patients with NF2-associated VS, with radiographic tumor responses reported in approximately 30-40% of patients and hearing improvement in 40-50% of cases [56–58]. However, these responses are variable, and severe adverse effects include hypertension and proteinuria limit the durability of this drug [57–60]. Notably, bevacizumab does not directly address tumor stiffness or mechanotransduction pathways during VS progression. Using VP to inhibit YAP activity, our findings demonstrate that mechanical confinement induces a YAP-dependent survival state, creating a stiffness-associated therapeutic vulnerability that is not apparent in conventional growth-based assays. These results highlight mechanotransduction as a potential therapeutic axis in VS.

Importantly, stiffness-dependent YAP signaling and fibrotic remodeling emerge as interconnected, rather than isolated, features of VS progression. Fibrotic remodeling is a defining feature of VS progression and is associated with increased firmness, hearing loss, and surgical difficulty [4, 5, 15]. In solid tumors, fibrosis reflects active ECM remodeling rather than collagen accumulation alone, driven by matrix reorganization through adhesion-dependent signaling [6, 7]. Matrix metalloproteinases, including MMP-2, MMP-9, and MMP-14, contribute to fibrotic tumor progression through ECM remodeling, while FAK signaling links matrix stiffness to cell–matrix adhesion and force transmission in mechanically reinforced tissues [7, 10, 45]. In our hydrogel model, increasing stiffness was accompanied by elevated MMP-9 and FAK activity which contrasts with the confinement-driven activation of mechanosensitive pathways observed in agarose. Fibrosis has been shown to be further driven by cytokines such as TGF-β which activate transcriptional programs that regulate ECM remodeling and collagen organization [11]. Consistent with this, TGF-β promoted collagen reorganization without altering bulk stiffness in our hydrogels. By recapitulating key features of fibrotic VS progression, this work reveals how biomechanical and biochemical cues converge to influence schwannoma behavior.

Antifibrotic strategies have shown variable efficacy in VS. In a CPA VS mouse model, treatment with losartan, an angiotensin receptor blocker, reduced collagen I and hyaluronan deposition, attenuated IL-6/STAT3 and TGF-β–associated inflammatory signaling and prevented tumor-induced hearing loss in mice [17]. Retrospective analyses reported associations between losartan use and hearing preservation in hypertensive VS patient cohorts [17, 61]. However, a separate study of 79 patients, including 29 treated with losartan, found no significant differences in tumor growth or hearing outcomes [62]. These mixed results suggest that targeting fibrosis may be insufficient in VS. In our model, inhibition of YAP-mediated mechanotransduction under TGF-β–induced fibrotic conditions attenuated profibrotic signaling, supporting a role for biomechanical crosstalk in fibrotic progression. Therefore, these findings highlight the need to target stiffness-dependent signaling in VS.

There are several limitations to this study. While primary VS cells were used to validate key mechanosensitive responses, their use across a broader range of functional assays, including cell proliferation, mechanical stress relief, and responses to pharmacologic treatment, represent important next steps. In addition, although collagen-based models capture adhesion-dependent signaling and matrix remodeling, they do not include other ECM components present in VS, such as hyaluronan and fibronectin, which may further influence stiffness-dependent schwannoma behavior. Furthermore, while stiffness was matched across hydrogel systems, pore size and the degree of confinement were not independently controlled and may contribute to the observed cellular responses. Published values indicate that collagen matrices in this concentration range exhibit pore sizes of approximately 800–3700 nm, whereas agarose gels of similar stiffness exhibit pore sizes on the order of 100–600 nm, suggesting that differences in pore architecture may also contribute to the observed phenotypes [63, 64]. Future studies directly measuring or independently controlling pore size will further clarify how stiffness and pore size together contribute to cellular confinement. Finally, findings from *in vitro* hydrogel systems require *in vivo* validation in animal models of VS to assess how host-derived cues and tissue-level constraints shape schwannoma progression.

Despite these limitations, mechanotransduction and fibrotic remodeling in VS are associated with poor clinical outcomes but are not captured in conventional in vitro models. These findings highlight the importance of this novel 3D system and support future drug screening and clinical trials, with the potential to identify targets that improve treatment success.

## Conclusion

In summary, this study establishes two complementary 3D hydrogel models that decouple mechanical confinement from adhesive matrix remodeling to interrogate mechanosensitive regulation in VS. By isolating confinement-driven responses from adhesion-dependent fibrotic processes, our findings demonstrate that VS adopt distinct mechanical adaptation states depending on ECM composition. These models recapitulate stiffness-dependent stress tolerance and fibrotic matrix remodeling in VS. Existing VS studies have not examined tumor behavior within tunable 3D mechanical environments, limiting understanding of how biomechanics influence disease progression. Thus, this work provides a versatile framework for studying how matrix mechanics shape tumor behavior and influence stiffness-associated therapeutic vulnerabilities in VS.

## Supplementary Information

Below is the link to the electronic supplementary material.

Supplementary file1 (PDF 3252 kb)

## Data Availability

All data and supporting information are available upon request to the authors.
