# Supplementary material for: Decoupling Mechanical Confinement and Fibrotic Extracellular Matrix Signaling in Vestibular Schwannoma Using Tunable 3D Hydrogels
Source: Cell Mol Bioeng. 2026 May 15;19(3):311–26. doi: 10.1007/s12195-026-00911-3 (PMC13365291; doi:10.1007/s12195-026-00911-3)
Supplement: Supplementary file 1 — Supplementary file1 (PDF 3252 kb) [file 12195_2026_911_MOESM1_ESM.pdf]

Decoupling Mechanical Confinement and Fibrotic Extracellular Matrix Signaling in Vestibular Schwannoma Using Tunable 3D Hydrogels

Melanie Fisher[1], Han TN. Nguyen[1], Rinky Ghosh[2], Yael Vodovotz[2] and Yin Ren[1]

[1]Division of Otology, Neurotology, and Cranial Base Surgery, Department of Otolaryngology - Head and Neck Surgery, The Ohio State University Wexner Medical Center, Columbus, OH

[2]Department of Food Science and Technology, The Ohio State University College of Food, Agricultural and Environmental Sciences, Columbus, OH

Corresponding Author:

Yin Ren, MD PhD

Division of Otology, Neurotology and Cranial Base Surgery

Department of Otolaryngology – Head and Neck Surgery

The Ohio State University Wexner Medical Center, Columbus, Ohio, USA

Email: Yin.Ren@osumc.edu

Supplementary Information

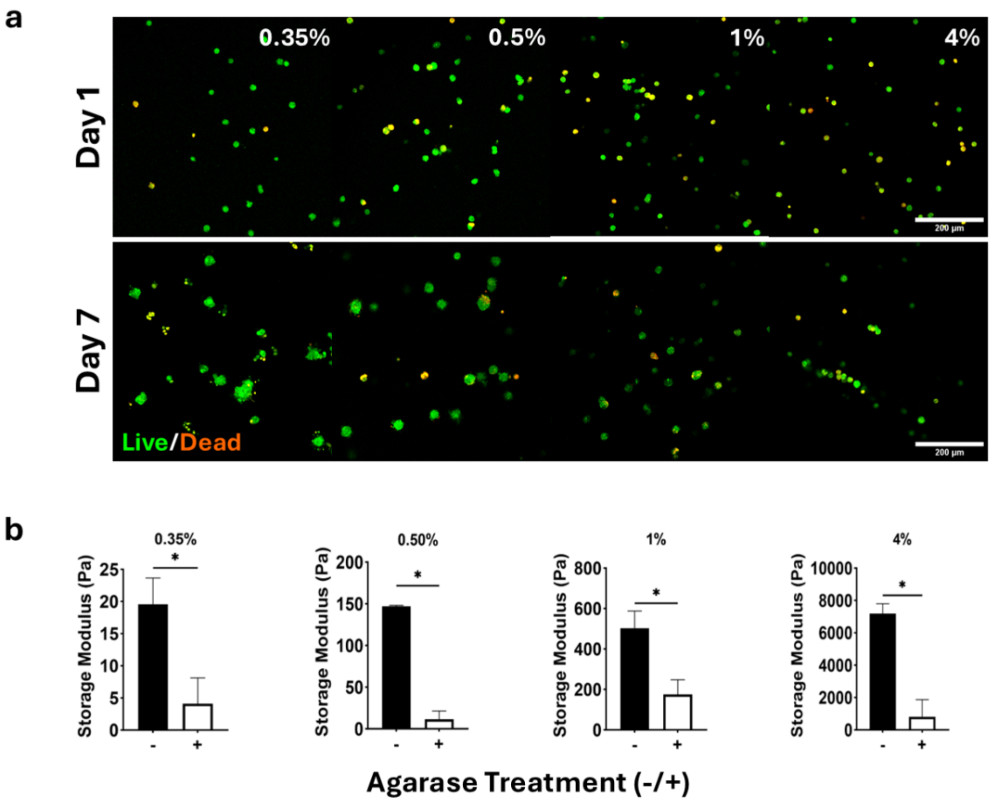

Fig. S1 Cell viability and agarase-mediated stiffness reduction in 3D agarose hydrogels

a Live/Dead staining using acridine orange and propidium iodide (AOPI) of HEI193 cells embedded in 3D agarose hydrogels of increasing concentrations (0.35–4%) at Day 1 and 7. Live cells are shown in green and dead cells in red. Scale bars are 200 μm. **b** Storage modulus (Pa) of agarose hydrogels (0.35–4%) before (−) and after (+) enzymatic digestion with agarase. N=3 hydrogels, $*p<0.05$ by Mann–Whitney test

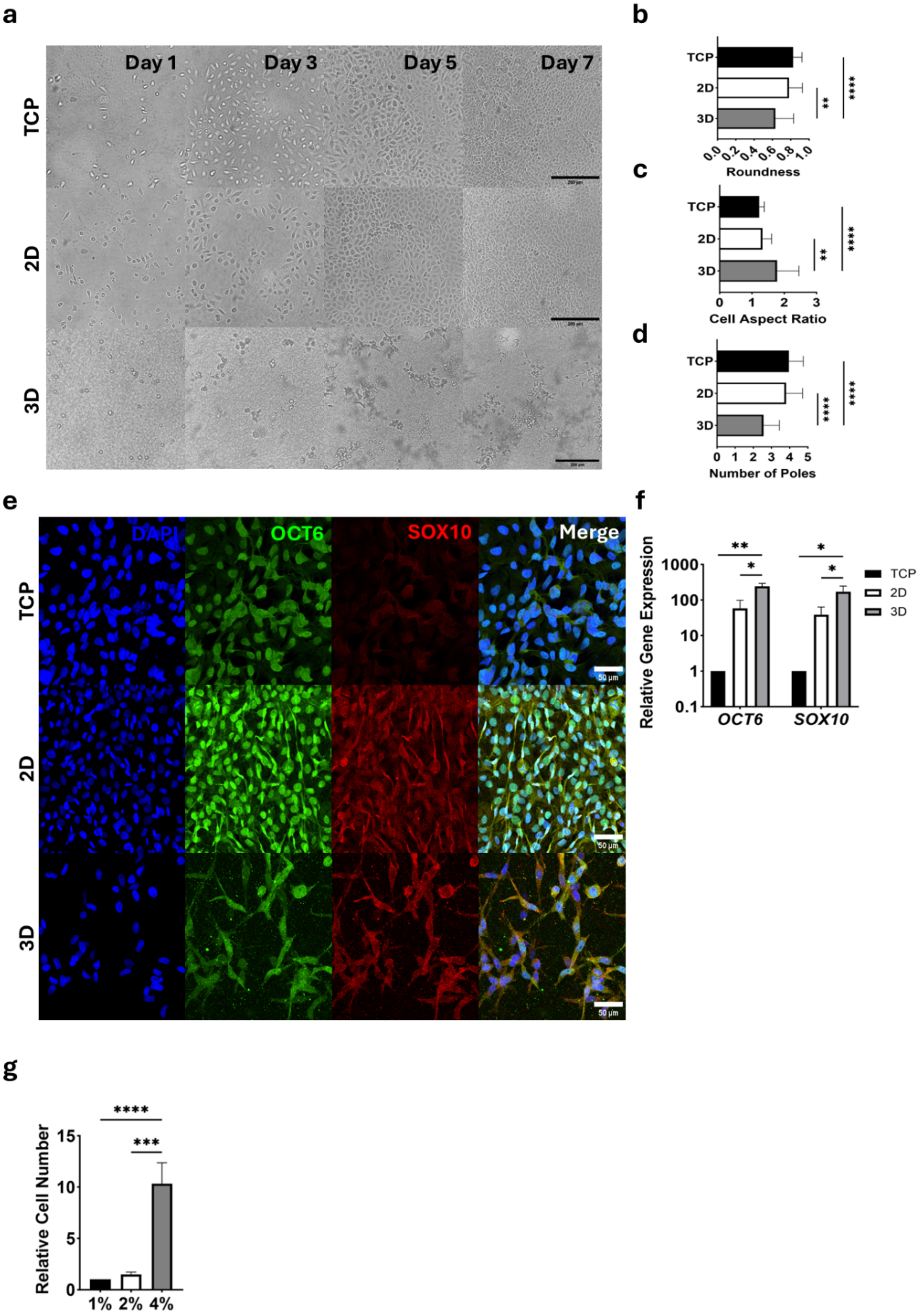
a
Day 1
Day 3
Day 5
Day 7
TCP
2D
3D
b
TCP
2D
3D
**
****
0.0 0.2 0.4 0.6 0.8 1.0
Roundness
c
TCP
2D
3D
**
****
0 1 2 3
Cell Aspect Ratio
d
TCP
2D
3D
****
****
0 1 2 3 4 5
Number of Poles
e
DAPI
OCT6
SOX10
Merge
TCP
2D
3D
50 µm
f
Relative Gene Expression
1000
100
10
1
0.1
**
*
*
*
OCT6
SOX10
TCP
2D
3D
g
****
***
Relative Cell Number
15
10
5
0
1%
2%
4%

Fig. S2 Effects of ECM composition and dimensionality on Schwannoma cell morphology and gene expression

a Representative brightfield images of HEI193 cells cultured on tissue culture plastic (TCP), 2D collagen substrate, or 3D collagen hydrogels (2 mg/mL) over 7 days. Scale bars are 200 µm. **b–d** Quantification of cell shape roundness **(b)**, aspect ratio **(c)**, and number of cellular poles **(d)** across culture conditions at Day 7. Roundness values closer to 1 indicate more circular morphology, whereas lower values indicate increased elongation. Higher aspect ratios and reduced pole number reflect elongated, Schwann-like morphology. **p<0.01, ****p<0.0001 by one-way ANOVA with Tukey's post hoc. **e** Representative immunofluorescence of HEI193 cells cultured on TCP, 2D collagen, and 3D collagen hydrogels stained for nuclei (DAPI, blue), OCT6 (green), and SOX10 (red). Scale bars are 50 µm. **f** qRT-PCR quantification of *OCT6* and *SOX10* mRNA relative expression across 2D and 3D collagen (normalized to TCP). *p<0.05, **p<0.01 by one-way ANOVA with Tukey's post hoc. **g** Relative cell number at Day 3 for HEI193 cells in 2D collagen-agarose composite gels containing 2 mg/mL collagen and increasing agarose concentrations (1–4%), normalized to the 1% agarose condition. N=3 hydrogels, ***p<0.001, ****p<0.0001 by one-way ANOVA with Tukey's post hoc.

a

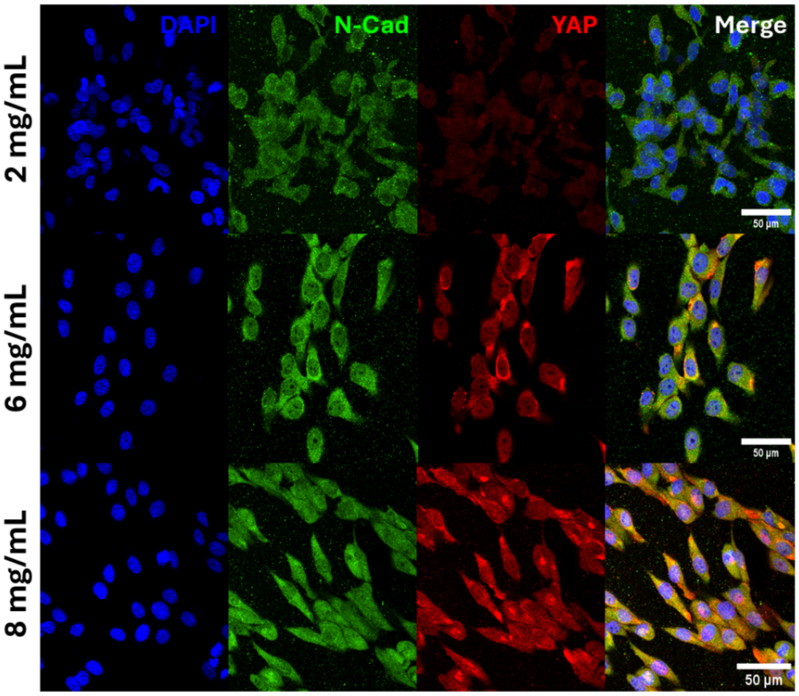
DAPI
N-Cad
YAP
Merge
2 mg/mL
6 mg/mL
8 mg/mL
50 μm
50 μm
50 μm

b

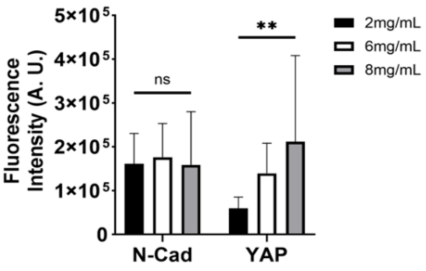
Fluorescence Intensity (A. U.)
5×10^5
4×10^5
3×10^5
2×10^5
1×10^5
0
ns
**
N-Cad
YAP
2mg/mL
6mg/mL
8mg/mL

c

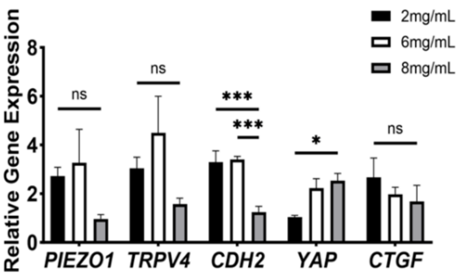
Relative Gene Expression
8
6
4
2
0
ns
ns
***
***
*
ns
PIEZO1
TRPV4
CDH2
YAP
CTGF
2mg/mL
6mg/mL
8mg/mL

d

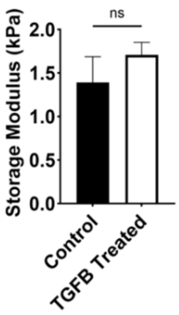
Storage Modulus (kPa)
2.0
1.5
1.0
0.5
0.0
ns
Control
TGFB Treated

Fig. S3 Fig. S3 N-cadherin and YAP expression, mechanosensitive gene expression, and TGF-β–mediated stiffness measurement in 3D collagen hydrogels

a–b Representative immunofluorescence images **(a)** and quantification **(b)** of N-cadherin and YAP in HEI193 cells in 3D collagen hydrogels of increasing stiffness. ns, not significant, $^{**}p<0.01$, by one-way ANOVA with Tukey's post hoc. Scale bars are 50 μm. **c** qRT-PCR quantification of normalized mRNA expression of *PIEZO1, TRPV4*, *CDH2*, YAP, and *CTGF* in HEI193 cells in 3D collagen hydrogels, normalized to stiffness-matched 2D collagen gels. ns, not significant, $^{*}p<0.05$, $^{***}p<0.001$, by one-way ANOVA with Tukey's post hoc. **d** Storage modulus (Pa) of collagen hydrogels with and without TGF-β treatment for 7 days. n=3 hydrogels, ns by Mann–Whitney U test

Gene Name	Primer	Sequence (5’- 3’)
**YAP**	Forward	TGTCCCAGATGAACGTCACAGC
	Reverse	TGGTGGCTGTTTCACTGGAGCA
**CTGF**	Forward	CTTGCGAAGCTGACCTGGAAGA
	Reverse	CCGTCGGTACATACTCCACAGA
**PIEZO1**	Forward	CATCTTGGTGGTCTCCTCTGTCT
	Reverse	CTGGCATCCACATCCCTCTCATC
**TRPV4**	Forward	CTACGCTTCAGCCCTGGTCTC
	Reverse	GCAGTTGGTCTGGTCCTCATTG
**FAK**	Forward	GCCTTATGACGAAATGCTGGGC
	Reverse	CCTGTCTTCTGGACTCCATCCT
**SOX10**	Forward	CCTCACAGATCGCCTACACC
	Reverse	CATATAGGAGAAGGCCGAGTAGA
**OCT6**	Forward	GCTCGAGAGCCACTTTCTCA
	Reverse	GTCATGCGCTTCTCCTTCTG
**SMAD3**	Forward	TGAGGCTGTCTACCAGTTGACC
	Reverse	GTGAGGACCTTGTCAAGCCACT
**SMAD7**	Forward	TGTCCAGATGCTGTGCCTTCCT
	Reverse	CTCGTCTTCTCCTCCCAGTATG
**TGFB**	Forward	TACCTGAACCCGTGTTGCTCTC
	Reverse	GTTGCTGAGGTATCGCCAGGAA
**LOX**	Forward	CTTGCACGTTTCCAATCGCA
	Reverse	GGCAGTGTCTGGAGTGAAGG
**TIMP1**	Forward	GGAGAGTGTCTGCGGATACTTC
	Reverse	GCAGGTAGTGATGTGCAAGAGTC
**TIMP2**	Forward	ACCCTCTGTGACTTCATCGTGC
	Reverse	GGAGATGTAGCACGGGATCATG
**TIMP3**	Forward	TACCGAGGCTTCACCAAGATGC
	Reverse	CATCTTGCCATCATAGACGCGAC
**TIMP4**	Forward	CACTACCATCTGAACTGTGGCTG
	Reverse	GCTTTCGTTCCAACAGCCAGTC
**MMP2**	Forward	CCCCAAAACGGACAAAGAG
	Reverse	CACGAGCAAAGGCATCATCC
**MMP9**	Forward	CACTGTCCACCCCTCAGAGC
	Reverse	GCCACTTGTCGGCGATAAGG
**MM14**	Forward	CGCTACGCCATCCAGGGTCTCAAA
	Reverse	CGGTCATCATCGGGCAGCACAAAA
**COL1A1**	Forward	GATTCCCTGGACCTAAAGGTGC
	Reverse	AGCCTCTCCATCTTTGCCAGCA
**COL4A1**	Forward	CAGGCACCCCATCTGTTGAT
	Reverse	CATTGCCCTGCACGTAGAGC

**COL6A1**	Forward	ACAGTGACGAGGTGGAGATCA
	Reverse	GATAGCGCAGTCGGTGTAGG
**CDH2**	Forward	CCTCCAGAGTTTACTGCCATGAC
	Reverse	GTAGGATCTCCGCCACTGATTC
**GAPDH**	Forward	GGATTTGGTCGTATTGGG
	Reverse	GGAAGATGGTGATGGGATT

Table S1. Forward and reverse primer sequences used for human RT-qPCR analysis

Supplemental Methods

2D Collagen-Agarose Composite Hydrogel Fabrication

Composite substrates were generated using neutralized FibriCol type I bovine collagen at a constant concentration of 2 mg/mL combined with agarose at final concentrations of 1%, 2%, or 4%. Neutralized collagen solutions were maintained at low temperature prior to mixing, while agarose was prepared as described above and equilibrated to approximately 37 °C. Neutralized collagen and agarose solutions were combined with gentle mixing to ensure homogeneity prior to dispensing onto tissue culture plastic. Composite substrates were allowed to partially set at room temperature for approximately 10 minutes, followed by incubation at 37 °C for 60 minutes to allow complete gelation. After gel formation, cells were seeded directly onto the gel surface with complete culture medium.

Brightfield Imaging and Cell Morphology Analysis

HEI193 cells were cultured on tissue culture plastic (TCP), two-dimensional (2D) collagen substrates, or embedded within three-dimensional (3D) collagen hydrogels prepared at 2 mg/mL at a seeding density of 10,000 cells per condition. Brightfield images were acquired at Days 1, 3, 5, 7 using a widefield microscope under identical imaging conditions across all culture platforms. Images were converted to grayscale and analyzed using Fiji (ImageJ). Using the ROI Manager tool, 25 cells were analyzed per condition at Day 7. Cell boundaries were defined by thresholding, and standard ImageJ shape descriptor function was used for morphology quantification (roundness and aspect ratio). Roundness values range from 0 to 1, with values closer to 1 indicating a more circular morphology. Aspect ratio was defined as the ratio of the major axis to the minor axis, with higher values reflecting increased elongation. The number of cellular poles was manually quantified by counting distinct protrusive extensions per cell, with polarity scores ranging from 2 (elongated, spindle-like morphology) to 5 (rounded morphology).
